# Physical activity and impaired left ventricular relaxation in middle aged adults

**DOI:** 10.1038/s41598-018-31018-z

**Published:** 2018-08-20

**Authors:** Seungho Ryu, Yoosoo Chang, Jeonggyu Kang, Kyung Eun Yun, Hyun-Suk Jung, Chan-Won Kim, Juhee Cho, Joao A Lima, Ki-Chul Sung, Hocheol Shin, Eliseo Guallar

**Affiliations:** 1 Department of Occupational and Environmental Medicine, Kangbuk Samsung Hospital, Sungkyunkwan University School of Medicine, Seoul, South Korea; 2 Center for Cohort Studies, Total Healthcare Center, Kangbuk Samsung Hospital, Sungkyunkwan University School of Medicine, Seoul, South Korea; 3 Department of Clinical Research Design and Evaluation, SAIHST, Sungkyunkwan University, Seoul, South Korea; 4 Division of Cardiology, Johns Hopkins University School of Medicine, Baltimore, Maryland USA; 5 Division of Cardiology, Department of Internal Medicine, Kangbuk Samsung Hospital, Sungkyunkwan University School of Medicine, Seoul, South Korea; 6 Department of Family Medicine, Kangbuk Samsung Hospital, Sungkyunkwan University School of Medicine, Seoul, South Korea; 7 Departments of Epidemiology and Medicine and Welch Center for Prevention, Epidemiology, and Clinical Research, Johns Hopkins University Bloomberg School of Public Health, Baltimore, Maryland USA

## Abstract

The aim of this study was to examine the relationship between physical activity level and impaired left ventricular (LV) relaxation in a large sample of apparently healthy men and women. We conducted a cross-sectional study in 57,449 adults who underwent echocardiography as part of a comprehensive health examination between March 2011 and December 2014. Physical activity level was assessed using the Korean version of the International Physical Activity Questionnaire Short Form. The presence of impaired LV relaxation was determined based on echocardiographic findings. Physical activity levels were inversely associated with the prevalence of impaired LV relaxation. The multivariable-adjusted odds ratios (95% confidence interval) for impaired LV relaxation comparing minimally active and health-enhancing physically active groups to the inactive group were 0.84 (0.77–0.91) and 0.64 (0.58–0.72), respectively (P for trend < 0.001). These associations were modified by sex (p for interaction <0.001), with the inverse association observed in men, but not in women. This study demonstrated an inverse linear association between physical activity level and impaired LV relaxation in a large sample of middle-aged Koreans independent of potential confounders. Our findings suggest that increasing physical activity may be independently important in reducing the risk of impaired LV relaxation.

## Introduction

Heart failure is a major public health problem that affects more than 5.8 million people in the United States and more than 23 million people worldwide and it results in significant morbidity, mortality and healthcare expenditures^1–4^. Several epidemiological studies have reported that nearly half of subjects with heart failure have left ventricular (LV) diastolic dysfunction^5–7^. Recent studies have described a rise in the proportion of heart failure with preserved LV ejection fraction relative to heart failure with reduced LV ejection fraction in recent decades^8,9^. Because of the serious consequences of overt heart failure, there is substantial interest in identifying potentially modifiable risk factors that may prevent or reduce the progression of LV diastolic dysfunction. Aging, diabetes, hypertension, obesity, connective tissue disease, exercise training are risk factors known to affect LV diastolic function^10–12^.

Especially, physical activity has multiple beneficial cardiovascular effects^13,14^ and is associated with reduced incidence of and lower mortality from cardiovascular disease^15,16^. Recent epidemiological studies have reported a close link between LV diastolic dysfunction and metabolic disorders including obesity, metabolic syndrome and insulin resistance^11,17^, all of which are also related to reduced physical activity. Most studies of physical activity and LV dysfunction, however, have focused on small cohorts of young male endurance athletes^14,18,19^ or of patients with overt heart disease^20–23^.

Impaired LV relaxation is likely to go ahead of LV chamber stiffness or systolic dysfunction during the development of heart failure and has been assigned as a sensitive sign of LV diastolic dysfunction^24^. Despite the fact that exercise is associated with better LV diastolic dysfunction, the relationship between physical activity and impaired LV relaxation remains largely unexplored in general population cohorts. Therefore, the aim of this study was to examine the association between physical activity and impaired LV relaxation in a large sample of Korean men and women free of clinical cardiovascular disease.

## Methods

### Study population

The Kangbuk Samsung Health Study was a cohort study of Korean men and women who attended a comprehensive annual or biennial health exam at the Kangbuk Samsung Hospital Total Healthcare Centers in Seoul and Suwon, South Korea^25^. The study population was composed of 61,775 participants who completed a physical activity questionnaire and underwent echocardiography as part of a comprehensive screening examination from March, 2011 to December, 2014.

We excluded 4,326 participants with one or more of the following exclusion criteria: self-reported history of malignancy (N = 1,495); self-reported history of cardiovascular disease (N = 537); presence of echocardiographic abnormalities, including ejection fraction <50%, hypertrophic or dilated cardiomyopathy, ischemic cardiomyopathy, valvular replacement or valvular surgery, mitral stenosis or regurgitation, atrial fibrillation and congenital disease (N = 2,218). Because some exclusion criteria overlapped, the total number of patients eligible for the present study was 57,449.

The Institutional Review Board of the Kangbuk Samsung Hospital approved this study. The requirement for informed consent was waived as we used only anonymized retrospective data routinely collected during the health screening process.

### Measurements

All examinations were conducted at the Kangbuk Samsung Hospital Total Healthcare Centers clinics in Seoul and Suwon. Data on demographic characteristics, medication use, medical history, smoking, alcohol consumption and education level were collected by self-administered, standardized questionnaires as previously described^25^.

We measured physical activity levels using the validated Korean version of the International Physical Activity Questionnaire (IPAQ) Short Form^26,27^, as previously described^25^. IPAQ Short Form measures the frequency and duration of walking or any other moderate to vigorous physical activity undertaken for more than 10 continuous minutes across all contexts (i.e., work, home and leisure) during a seven-day period. Physical activity levels were classified into three categories: inactive, minimally active (600 MET-minutes per week), and health-enhancing physically active (HEPA; 3000 MET-minutes per week)^25^. The total amount of weekday sitting time was assessed using the following single question: “During the last seven days, how much time did you usually spend sitting on a weekday?”^26^. The sitting time was divided into the following categories commonly found in previous studies: <5, 5–9, and ≥10 hours/day^28^.

The Pittsburgh Sleep Quality Index (PSQI) was used to assess sleep quality^29^. We used component 3 of the PSQI, which is concerned with the number of hours of actual nighttime sleep obtained during the past month, to assess sleep duration. We measured usual dietary intake using a 103-item, self-administered food frequency questionnaire designed and validated for use in Korea^30^. We calculated total energy and nutrient intake with a food composition tables developed by the Korean Nutrition Society^30^.

Trained nurses measured height and weight while the participants wore a lightweight hospital gown and no shoes. Body mass index (BMI) was calculated as weight divided by height squared (kg/m^2^). Obesity was defined as BMI >25.0 kg/m^2^ according to the criteria proposed for Asian populations^31^. Blood pressure was measured using an automated oscillometric device (53000, Welch Allyn, New York, USA) with the participants in a sitting position with the arm supported at heart level. Hypertension was defined as a systolic blood pressure ≥140 mmHg, a diastolic blood pressure ≥90 mmHg, or current use of antihypertensive medication.

Measurements for serum biochemical parameters, including total cholesterol, triglycerides, high-density lipoprotein cholesterol (HDL-C), low-density lipoprotein cholesterol (LDL-C), insulin, glucose, hemoglobin A1c, and high-sensitivity C reactive protein (hsCRP) are described in detail elsewhere^25^.

### Echocardiography

Conventional echocardiography was performed with ultrasound scanners (Vivid 7 and E9, General Electric, Milwaukee, WI, USA) by trained sonographers following a standardized protocol^32^. Linear measurements of intraventricular septum thickness (IVST), left posterior wall thickness (PWT) and diameter of the left ventricular cavity at the end of diastole and systole were obtained in M-mode in the parasternal long axis view. LV mass in grams was calculated using the following equation: LV mass = 0.8 × [1.04 × (LVEDD + IVST + PWT)^3^ − LVEDD^3^] + 0.6^33,34^. Left ventricular mass index (LVMI) was determined as LV mass/body surface area (g/m^2^). The anteroposterior diameter of the left atrium (LA) was measured in all participants.

Diastolic function was evaluated using pulse-wave Doppler transmitral LV inflow in an apical four-chamber view. Early diastolic mitral inflow peak velocity (E), late diastolic peak velocity (A) during atrial contraction, and deceleration time of the E velocity were also measured. The early (E’) and late (A’) tissue velocities were obtained from Tissue Doppler imaging of the septal mitral annuls. Since E’ is primarily determined by myocardial relaxation and to a lesser extent by restoring forces and filling pressures, impaired LV relaxation was defined based on decreased E’(<7 cm/s) in this study^35^. In this study, only 102 participants (0.18%) had increased E/E’(>15).

### Statistical analyses

The characteristics of the study participants were explored using physical activity categories. Mean values (95% confidence intervals [CIs]) of echocardiographic parameters were also estimated using physical activity categories. We conducted a logistic regression to estimate the odds ratios (with 95% CIs) for impaired LV relaxation by comparing the categories of physical activity with the reference category. We used three models with progressive adjustments. Model 1 was adjusted for age, sex, study center (Seoul, Suwon), and year of screening exam (one-year categories). Model 2 further included alcohol intake (0, <20, ≥20 g/d, or unknown), smoking (never, past, current, or unknown), educational level (high school graduate or less, community college or university graduate, graduate school or higher, and unknown), total calorie intake (quintile of total calorie intake or missing), sleep duration, family history of heart disease, history of hypertension and history of diabetes. Model 3 further adjusted for BMI, HOMA-IR, hsCRP, blood pressure and LVMI. To test for linear trends, we use the category rank as a continuous variable in the regression models. In addition, we examined the relationship between sitting time and impaired LV relaxation.

We performed stratified analyses in clinically relevant subgroups defined by sex (female vs male), age (<50 vs ≥50 years), smoking (never or ex-smoker vs current smoker), alcohol intake (<20 vs ≥20 g of alcohol per day), BMI (<25 vs ≥25 kg/m^2^), diabetes (no vs yes) and hypertension (no vs yes). Interactions between subgroups were tested using likelihood ratio tests to compare models with and without product terms.

All P-values were two-tailed, and P values < 0.05 were considered statistically significant. We used STATA version 14.0 (Stata Corp., College Station, TX, USA) for data analysis.

## Results

The mean (SD) age, BMI, physical activity, and sitting time of study participants were 40.4 (7.6) years, 24.0 (3.2) kg/m^2^, 1,549.5 (3,257.9) METs, and 8.0 (3.7) hours, respectively. The correlation between physical activity level (MET) and sitting time was −0.14 (P < 0.001). The proportions of inactive, minimally active and HEPA participants were 45.0, 38.8, and 16.2%, respectively. Compared to inactive participants, those with HEPA were more likely to be older, and obese, more likely to have a history of diabetes and hypertension, more likely to have higher total calorie intake, alcohol intake, systolic and diastolic blood pressure, glucose and HDL-C levels, less likely to be smokers, and more likely to have lower levels of education total cholesterol, LDL-C, triglycerides, HOMA-IR and sleep duration (Table 1).

**Table 1:** Baseline characteristics of the study participants according to physical activity level.

Characteristics	Overall	Physical activity			P for trend
		Inactive	Minimally active	HEPA	
Number	57,449	25,865	22,261	9,323	
Men (%)	75.8	72.2	80.4	74.6	<0.001
Age (years)^a^	40.4 (7.6)	40.0 (7.1)	40.4 (7.6)	41.6 (8.7)	<0.001
BMI (kg/m^2^)^a^	24.0 (3.2)	23.8 (3.3)	24.0 (3.1)	24.2 (3.1)	<0.001
Seoul center (%)	56.3	55.0	57.3	57.4	0.001
Obesity (%)	34.5	33.5	35.0	36.1	<0.001
Current smoker (%)	24.9	25.3	25.2	23.1	<0.001
Alcohol intake (%)^b^	25.7	24.7	25.4	29.4	<0.001
High education level (%)^c^	86.3	86.1	89.2	80.0	<0.001
Family history of CVD (%)	6.4	6.4	6.2	6.8	0.441
History of diabetes (%)	10.9	2.8	3.0	4.0	<0.001
History of hypertension (%)	3.1	9.8	11.0	13.3	<0.001
Systolic BP (mmHg)^a^	111.0 (12.8)	110.1 (12.9)	111.5 (12.6)	112.2 (12.7)	<0.001
Diastolic BP (mmHg)^a^	72.3 (10.2)	72.0 (10.4)	72.6 (10.1)	72.2 (10.1)	0.002
Glucose (mg/dL)^a^	96.5 (15.3)	96.2 (15.7)	96.8 (15.1)	96.9 (14.7)	<0.001
Total cholesterol (mg/dL)^a^	198.0 (34.5)	198.1 (34.8)	198.5 (34.3)	196.7 (33.8)	0.015
LDL-C (mg/dL)^a^	124.3 (31.6)	124.2 (32.0)	125.0 (31.3)	122.5 (31.3)	0.006
HDL-C (mg/dL)^a^	55.6 (14.3)	55.1 (14.3)	55.1 (13.9)	57.8 (14.9)	<0.001
Triglycerides (mg/dL)^d^	105 (73–156)	109 (75–160)	106 (75–157)	95 (67–138)	<0.001
HOMA-IR^d^	1.32 (0.87–1.96)	1.36 (0.90–2.03)	1.32 (0.87–1.94)	1.17 (0.77–1.78)	<0.001
hsCRP (mg/L)^d^	0.5 (0.3–1.0)	0.5 (0.3–1.0)	0.5 (0.3–0.9)	0.4 (0.3–0.9)	<0.001
Sleep duration (h/day)^a^	6.38 (1.02)	6.40 (1.05)	6.38 (0.99)	6.36 (1.02)	0.001
Total energy intake (kcal/d) ^d,e^	1554.8 (1218.4–1933.0)	1509.9 (1174.9–1876.9)	1591.7 (1264.9–1972.4)	1597.0 (1241.3–1989.4)	<0.001

Data are ^a^mean (standard deviation), ^d^median (interquartile range), or percentage.

Abbreviations: BMI, body mass index; BP, blood pressure; CVD, cardiovascular disease; HDL-C, high-density lipoprotein cholesterol; HOMA-IR, homeostasis model assessment of insulin resistance.

^b^ ≥ 20 g/day; ^c^ ≥ College graduate; ^e^among 40,298 participants with plausible estimated energy intake levels (within three standard deviations from the log-transformed mean energy intake).

In models adjusted for age, sex, center and year of screening exam, E, A, E/E’, E/A ratio, LVEDD, LV mass, LVMI and LA size were positively related to physical activity level whereas heart rate and septal E’ were inversely related to physical activity level (Table 2).

**Table 2:** Estimated^a^ mean values (95% CIs) of echocardiographic characteristics of the study participants by physical activity level.

Characteristics	Physical activity			P for trend
	Inactive	Minimally active	HEPA	
Number of participants	25,865	22,261	9,323	
Heart rate	65.5 (65.3–65.6)	64.5 (64.4–64.6)	62.3 (62.1–62.5)	<0.001
Ejection fraction	66.7 (66.6–66.8)	66.7 (66.6–66.7)	66.6 (66.5–66.8)	0.31
E (cm/s)	69.7 (69.2–70.1)	71.0 (70.5–71.5)	72.1 (71.3–72.8)	<0.001
A (cm/s)	52.5 (51.0–54.0)	54.0 (52.4–55.6)	52.6 (50.2–55.1)	<0.001
E/E'	7.42 (7.37–7.47)	7.49 (7.44–7.54)	7.53 (7.45–7.61)	<0.001
E/A ratio	1.41 (1.39–1.42)	1.42 (1.41–1.44)	1.44 (1.42–1.46)	<0.001
Septal E’ (cm/s)	10.7 (10.3–11.1)	10.7 (10.2–11.1)	10.6 (9.8–11.3)	<0.001
Septal A’ (cm/s)	8.9 (8.5–9.2)	8.9 (8.5–9.2)	8.6 (8.0–9.2)	0.26
LVEDD (mm)	48.7 (48.6–48.7)	49.0 (48.9–49.0)	49.8 (49.7–49.8)	<0.001
LV mass (g)	133.3 (133.0–133.7)	135.3 (134.9–135.6)	142.0 (141.5–142.6)	<0.001
LVMI (g/ BSA, g/m^2^)	73.3 (73.2–73.5)	74.4 (74.2–74.5)	77.8 (77.5–78.0)	<0.001
LA size (mm)	34.0 (33.7–34.2)	34.1 (33.8–34.4)	35.3 (34.9–35.7)	<0.001

^a^Adjusted for age, sex, center, and year of screening exam.

Early diastolic mitral inflow peak velocity (E), late diastolic peak velocity (A) during atrial contraction, and deceleration time of the E velocity were measured. The early (E’) and late (A’) tissue velocities were obtained from tissue Doppler imaging of the septal mitral annulus.

Abbreviations: BSA, body surface area; LV, left ventricular; LVEDD, left ventricular end-diastolic diameter; LVMI, left ventricular mass index.

The overall prevalence of impaired LV relaxation was 6.7%. After adjusting for age, sex, center and year of screening exam, the odds ratios (95% CIs) for impaired LV relaxation comparing minimally active and HEPA groups to the inactive group were 0.84 (0.78–0.91) and 0.68 (0.62–0.76), respectively (Table 3). Further adjustment for potential confounding variables did not materially alter the findings. When adjusted for potential intermediate variables, the association was still statistically significant. The respective odds ratios (95% CIs) for impaired LV relaxation comparing minimally active and HEPA groups to the inactive group were 0.84 (0.78–0.92) and 0.65 (0.58–0.73), respectively (P for trend < 0.001) (Table 3**)**. On the other hand, sitting time was not significantly associated with impaired LV relaxation (Appendix Table 1**)**. The interaction of physical activity and sitting time for impaired LV relaxation was not statistically significant (p for interaction = 0.36). The multivariable adjusted odds ratios (95% CIs) for impaired LV relaxation comparing HEPA groups to the minimally active group were 0.77 (0.69–0.86) (Appendix Table 2).

**Table 3:** Odds ratios^a^ (95% CIs) of impaired left ventricular relaxation by physical activity level.

Physical activity level	Number	Cases	Multivariate-adjusted OR^a^		
			Model 1	Model 2	Model 3
Inactive	25,865	1,742	1.00 (reference)	1.00 (reference)	1.00 (reference)
Minimally active	22,261	1,460	0.84 (0.78–0.91)	0.84 (0.77–0.91)	0.84 (0.78–0.92)
HEPA	9,323	644	0.68 (0.62–0.76)	0.64 (0.58–0.72)	0.65 (0.58–0.73)
*P for trend*			<0.001	<0.001	<0.001

^a^Estimated from logistic regression models. Multivariable Model 1 was adjusted for age, sex, center, and year of screening exam; Model 2: Model 1 plus adjustments for smoking status, alcohol intake, educational level, total calorie intake, sleep duration, family history of heart disease, history of diabetes, and history of hypertension; Model 3: Model 2 plus adjustments for BMI, HOMA-IR, systolic blood pressure, hsCRP, heart rate and LVMI (g/ BSA, g/m^2^).

**Figure 1:**
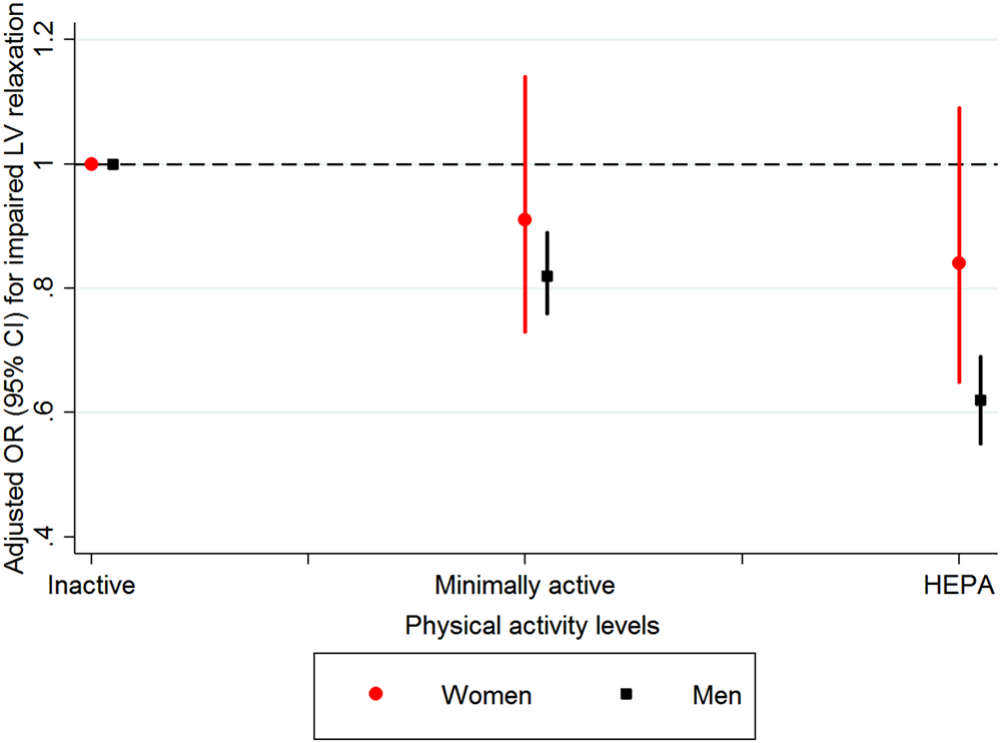
Multivariate-adjusted odds ratios^a^ (95% CIs) of impaired LV relaxation according to physical activity level in women and men. ^a^Estimated from a logistic regression model. The multivariable model was adjusted for age, sex, year of screening exam, center, educational level, alcohol intake, smoking status, total calorie intake, sleep duration, family history of heart disease, history of hypertension, and history of diabetes. The P-value for the interaction of sex and physical activity levels for impaired LV relaxation was < 0.001.

The association between physical activity and impaired LV relaxation was similar in pre-specified subgroups of study participants except those divided based on sex (Table 4 and Fig. 1). The association between physical activity and impaired LV relaxation was observed in men, but not in women (Fig. 1, P interaction < 0.001). The association between physical activity levels and impaired LV relaxation was still present in non-obese participants (BMI <25 kg/m^2^).

**Table 4:** Odds ratios^a^ (95% CIs) of impaired left ventricular relaxation according to physical activity level in clinically relevant subgroups.

Subgroup	Physical activity levels			P for trend	P for interaction
	Inactive	Minimally active	HEPA		
Sex					<0.001
Female (N = 13,912)	1.00 (reference)	0.88 (0.70–1.11)	0.85 (0.66–1.10)	0.196	
Male (N = 43,537)	1.00 (reference)	0.82 (0.76–0.89)	0.61 (0.55–0.69)	<0.001	
Age					0.073
<50 years (N = 51,642)	1.00 (reference)	0.83 (0.75–0.91)	0.62 (0.54–0.71)	<0.001	
≥50 years (N = 5,807)	1.00 (reference)	0.91 (0.79–1.04)	0.83 (0.71–0.97)	0.019	
Current smoker					0.695
No (N = 40,135)	1.00 (reference)	0.86 (0.78–0.95)	0.61 (0.54–0.70)	<0.001	
Yes (N = 13,310)	1.00 (reference)	0.81 (0.70–0.94)	0.62 (0.49–0.77)	<0.001	
Alcohol intake					0.081
<20 g/day (N = 41,254)	1.00 (reference)	0.85 (0.77–0.95)	0.69 (0.60–0.79)	<0.001	
≥20 g/day (N = 14,293)	1.00 (reference)	0.92 (0.72–0.94)	0.57 (0.48–0.68)	<0.001	
BMI					0.071
<25 kg/m^2^ (N = 37,538)	1.00 (reference)	0.85 (0.76–0.96)	0.70 (0.59–0.81)	<0.001	
≥25 kg/m^2^ (N = 19,769)	1.00 (reference)	0.83 (0.75–0.92)	0.60 (0.52–0.70)	<0.001	
Diabetes					0.601
No (N = 54,654)	1.00 (reference)	0.83 (0.77–0.91)	0.64 (0.57–0.72)	<0.001	
Yes (N = 2,794)	1.00 (reference)	0.82 (0.66–1.02)	0.66 (0.50–0.87)	0.002	
Hypertension					0.038
No (N = 49,123)	1.00 (reference)	0.85 (0.77–0.94)	0.67 (0.58–0.76)	<0.001	
Yes (N = 8,252)	1.00 (reference)	0.81 (0.71–0.92)	0.61 (0.52–0.72)	<0.001	

^a^Estimated from logistic regression. The multivariable model was adjusted for age, sex, center, year of screening exam, smoking status, alcohol intake, educational level, total calorie intake, sleep duration, family history of heart disease, history of diabetes, and history of hypertension.

## Discussion

There were several novel findings in this large sample of Korean men and women. First, we observed an inverse linear association between physical activity level and impaired LV relaxation independent of potential confounders. Both minimally active levels and HEPA were inversely associated with impaired LV relaxation. Second, these associations were modified by sex. The inverse association between physical activity and impaired LV relaxation was observed only for men. Lastly, the association between physical activity level and impaired LV relaxation was still observed in non-obese participants.

Many epidemiologic studies have reported an association of inadequate physical activity with diabetes, coronary heart disease, stroke and increased cardiovascular mortality^15,16,36^. However, the relationship between physical activity and impaired LV relaxation in general a population sample remains largely unexplored. Most studies of the cardiac effects of physical activity have focused on LV function among elderly patients with heart problems or in small cohorts of young male endurance athletes^14,18–23^. A small investigation of 36 healthy participants reported that a sedentary lifestyle was related to decreased LV compliance, leading to diminished diastolic performance^37^. To our knowledge, this is the first study to show an inverse linear association between physical activity and the prevalence of impaired LV relaxation in a large sample of adults who were not elite athletes or patients with heart failure.

The mechanism by which physical activity was inversely related to the prevalence of impaired LV relaxation has yet to be identified. Because obesity is associated with changes in cardiac structure and function, including LV diastolic dysfunction, adiposity might represent a possible mechanism linking physical activity with impaired LV relaxation^11,38,39^. In this study, adjusting for BMI did not change the inverse association between physical activity levels and impaired LV relaxation. In addition, an inverse relationship between physical activity and impaired LV relaxation was still observed even in participants with a BMI <23 kg/m^2^, the group in which confounding from BMI should be the smallest.

Insulin resistance might also be another possible mechanism to explain the association of physical activity with impaired LV relaxation. Physical activity leads to greater insulin sensitivity due to increases in the glucose transport protein-4 levels and muscle glycogen synthase activity, decreases in serum triglyceride concentrations and increases in the muscle capillary network^40^. Indeed, recent epidemiological studies have described a close link between LV diastolic dysfunction and insulin resistance or metabolic syndrome^17,41^. In our study, however, the relationship between physical activity and impaired LV relaxation remained statistically significant after further adjusting for HOMA-IR suggesting that physical activity may directly affect LV diastolic function through biological pathways independent of obesity or insulin resistance. The positive association between physical activity and impaired LV relaxation might be explained by high blood pressure. The relationship of hypertension with LV diastolic dysfunction has been established^42,43^. These results did not change after adjustment for history of hypertension and systolic blood pressures.

Prolonged endurance exercise leads to a balanced enlargement of ventricular mass and dimensions^14^, resulting in enhanced cardiac performance without an obvious change in contractility, which thus might explain the low prevalence of impaired LV relaxation among participants with minimally active levels or HEPA levels^37,44^. Furthermore, prolonged exercise could have beneficial effects due to the maintenance of vascular elasticity and thus smaller arterial load^37^. Endurance training preserves vascular elasticity with aging and therefore might prevent changes in cardiac adaptation, such as focal proliferation of matrix or alteration of myocyte morphology^45,46^.

In the current analyses, the association between physical activity levels and impaired LV relaxation was statistically significant in men, but not in women. Despite conflicting results on possible sex differences in the protective effects of physical activity, several investigations have shown that there is differential remodeling in responses to aerobic exercise by sex. Although the reasons for sex-dependent effects of physical activity on impaired LV relaxation are not fully understood, there could be strong genetic modifiers of cardiac adaptation and exercise capacity^47,48^. A recent experimental study showed that the α1-adrenergic system is very important in determining heart size and the ability of the heart to respond to pathological and physiologic stimuli only in male mice, and this sex difference remained following ovariectomy in females^49^. Sex-related difference could also be related to shorter stature, hence earlier wave reflection and diastolic dysfunction^50^. Further research is needed to understand this sex-related difference.

Our study had some limitations that should be considered in the interpretation of our findings. First, we don’t have any information related to cardiorespiratory fitness, and because physical activity was self-reported, measurement error was inevitable. Specifically, people often have trouble recalling light-intensity exercise and tend to misreport the amount of time spent doing in such activities^51^. Misclassification is likely but might be non-differential. As a consequence, the true association of physical activity with LV diastolic function could be much stronger than shown here. Second, the cross-sectional design in this study limited our ability to determine temporality and prove causal relationships. Third, we have no information about LA volume or peak tricuspide velocity among the measurements recommended by the American Society of Echocardiography to assess LV diastolic function^35^. Future prospective studies that assess all four variables: annular E’ velocity, E/E’ ratio, LA volume, and peak tricuspide velocity are needed to examine potential causal associations of physical activity with the development of LV diastolic dysfunction. Finally, participants in our study were highly educated, young and middle-aged Koreans who regularly underwent health check-up exams, most often as a part of work-related health screening programs; thus, the results from our study may not be generalizable to other populations with different demographic composition. Despite these potential limitations, major strengths of our study were its large sample size, a comprehensive inclusion of potential confounding variables, and the availability of high-quality imaging and laboratory procedures with extensive quality control measures.

## Conclusion

This study demonstrated an inverse linear association between physical activity level and impaired LV relaxation independently of potential confounders in a large sample of young and middle-aged Koreans. This association was shown primarily in men, but not in women. Our findings suggest that increasing physical activity may be independently associated with reduced risk of impaired LV relaxation.

## Electronic supplementary material

Supplementary Table
