# Supplementary material for: Physical activity and impaired left ventricular relaxation in middle aged adults
Source: Sci Rep. 2018 Aug 20;8:12461. doi: 10.1038/s41598-018-31018-z (PMC6102302; doi:10.1038/s41598-018-31018-z)
Supplement: Supplementary file 1 — Supplementary Table [file 41598_2018_31018_MOESM1_ESM.pdf]

Physical activity and impaired left ventricular relaxation in middle aged adults

Seungho Ryu[1,2,3], Yoosoo Chang[1,2,3], Jeonggyu Kang[2], Kyung Eun Yun[2], Hyun-Suk Jung[2], Chan-Won Kim[2], Juhee Cho[2,3], Joao A Lima[4], Ki-Chul Sung[5], Hocheol Shin[2,6], Eliseo Guallar[7]

[1] Department of Occupational and Environmental Medicine, Kangbuk Samsung Hospital, Sungkyunkwan University School of Medicine, Seoul, South Korea

[2] Center for Cohort Studies, Total Healthcare Center, Kangbuk Samsung Hospital, Sungkyunkwan University School of Medicine, Seoul, South Korea

[3] Department of Clinical Research Design and Evaluation, SAIHST, Sungkyunkwan University, Seoul, South Korea

[4] Division of Cardiology, Johns Hopkins University School of Medicine, Baltimore, Maryland, USA.

[5] Division of Cardiology, Department of Internal Medicine, Kangbuk Samsung Hospital, Sungkyunkwan University School of Medicine, Seoul, South Korea

[6] Department of Family Medicine, Kangbuk Samsung Hospital, Sungkyunkwan University School of Medicine, Seoul, South Korea

[7] Departments of Epidemiology and Medicine and Welch Center for Prevention, Epidemiology, and Clinical Research, Johns Hopkins University Bloomberg School of Public Health. Baltimore, Maryland, USA.

Appendix Table 1. Odds ratios[a] (95% CIs) of impaired left ventricular relaxation by sitting time

Sitting time	Number	Cases	Multivariate-adjusted OR[a]	
			Model 1	Model 2
<5 hours/day	10,550	950	1.06 (0.96–1.18)	1.08 (0.97–1.20)
5–9 hours/day	23,463	1,663	0.99 (0.91–1.07)	1.01 (0.93–1.10)
≥10 hours/day	23,436	1,251	1.00 (reference)	1.00 (reference)
P for trend			0.30	0.18

[a] Estimated from logistic regression models. Multivariable Model 1 was adjusted for age, sex, center, and year of screening exam; Model 2: Model 1 plus adjustments for smoking status, alcohol intake, educational level, total calorie intake, sleep duration, family history of heart disease, history of diabetes, history of hypertension, and physical activity level

Appendix Table 2. Odds ratios[a] (95% CIs) of impaired left ventricular relaxation by physical activity level

Physical activity level	Multivariate-adjusted OR[a]		
	Model 1	Model 2	Model 3
Minimally active	1.00 (reference)	1.00 (reference)	1.00 (reference)
HEPA	0.81 (0.73–0.90)	0.77 (0.69–0.86)	0.77 (0.68–0.86)

[a]Estimated from logistic regression models. Multivariable Model 1 was adjusted for age, sex, center, and year of screening exam; Model 2: Model 1 plus adjustments for smoking status, alcohol intake, educational level, total calorie intake, sleep duration, family history of heart disease, history of diabetes, and history of hypertension; Model 3: Model 2 plus adjustments for BMI, HOMA-IR, systolic blood pressure, hsCRP, heart rate and LVMI (g/ BSA, g/m^2)
